# Moral neutralization: Nurses’ evolution in unethical climate workplaces

**DOI:** 10.1186/s12910-020-00558-3

**Published:** 2020-11-17

**Authors:** Hamideh Hakimi, Soodabeh Joolaee, Mansoureh Ashghali Farahani, Patricia Rodney, Hadi Ranjbar

**Affiliations:** 1 Nursing Care Research Center (NCRC), School of Nursing and Midwifery, Iran University of Medical Sciences, Tehran, Iran; 2 UBC Centre for Health Evaluation and Outcome Sciences (CHÉOS), Vancouver, BC Canada; 3 School of Nursing, University of British Columbia, Vancouver, Canada; 4 Mental Health Research Center, Psychosocial Health Research Institute, Iran University of Medical Science, Tehran, Iran

**Keywords:** Morality, Nurses, Health care system, Ethical practice, Moral climate

## Abstract

**Introduction:**

Good quality of care is dependent on nurses’ strong clinical skills and moral competencies, as well. While most nurses work with high moral standards, the moral performance of some nurses in some organizations shows a deterioration in their moral sensitivity and actions. The study reported in this paper aimed to explore the experiences of nurses regarding negative changes in their moral practice.

**Materials and methods:**

This was a qualitative study utilizing an inductive thematic analysis approach, which was conducted from February 2017 to September 2019. Twenty-five nurses participated in semi-structured interviews.

**Results:**

The main theme that emerged from our analysis was one of moral neutralization in the context of an unethical moral climate. We found five sub-themes, including: (1) feeling discouraged; (2) normalization; (3) giving up; (4) becoming a justifier; and (5) moral indifference.

**Conclusions:**

Unethical moral climates in health organizations can result in deterioration of morality in nurses which can harm both patients and health systems. Some unethical behaviors in nurses can be explained by this process.

## Introduction

Providing high-quality ethical care is the main goal of the health system. Nursing ethical practice means that “the patient is the central focus of a nurse’s work and that they are to protect the moral environment in which patients receive care” [1]. Ethical practice in nursing aims to act following seven principles of non-maleficence, beneficence, health maximization, efficiency, respect for autonomy, justice, and proportionality [2]. To achieve this goal all actions should be based on recent evidence and professional guidelines, including moral rules and regulations [3]. Nurses are one of the main members of each health providing team, and their performance has a substantial effect on patient's health and safety [4]. The complexity of the nurse’s role requires the highest level of moral standards and performance [5]. Every action a nurse performs in the care process has a moral dimension [6]; and based on ICN and Iranian Codes of ethics all nurses' actions should be according to ethical codes [7, 8]. Nursing education is therefore not just about acquiring knowledge and learning practical skills, but also about gaining professional and ethical values [9]. The importance of ethics in care is to such an extent that it sometimes outweighs the technical aspects of nursing [10]; in this regard, any care should be provided ethically to have the highest quality. As nurses become more sensitive to the ethical aspects of care, the quality of care provided also tends to increase [11, 12]. Because the level of sensitivity to ethical issues and understanding the ethical dimensions of care are influenced by cultural and workplace conditions, and beliefs of nurses, the level of moral sensitivity of nurses can vary [13]. Nurses enter the nursing profession with a set of moral values, beliefs and competencies, and they are expected to develop ethical skills along with their clinical skills during their education and work.

Morality is a cornerstone concept of nursing practice and education. Nursing instructors usually face a challenge regarding the nurse’s moral practice. While they provide instruction about ethical codes and moral issues to nursing students, nurse’s moral practice transforms rapidly after they leave the educational context and begin to work in the real situation. As is the case with learning professional techniques, nurses' moral performance is affected by their work environment. To examine why these changes happen, we searched the relevant literature, and we found that changes in moral competencies were the topic of several studies [14–19].

More specifically, our review showed that there was a significant concentration on the development of morality in nursing students [20–24]. Furthermore, the components of moral practice, including sensitivity and courage were evaluated in several studies [25–30]. For example, in their study involving, [32] showed that nurses apply ethics knowledge in real work situations infrequently [11]. The results of other studies showed that sometimes deterioration happened in the moral practice of nurses. For example, the results of Dalvand et al. 2015 and [32] indicated that with an increase in the age of nurses, respect for patient autonomy was decreased [31, 32]. The results of another showed that individual character and responsibility, communication challenges, organizational preconditions, support systems, educational and cultural development affect nurses' professional ethics[33].

Moral evolution, changes in moral knowledge, and competencies, in nurses during their practice was assessed before mainly through quantitative approaches. Some qualitative studies also examined the changes and factors that may affect nurses'moral behaviors. The process of negative changes in nurses' conduct gains low attention in the research literature, despite the presence of various studies that reflect concerns about their unethical performance. While most nurses work with the highest level of moral standards, the moral performance of some nurses in some organizations shows a deterioration in their moral sensitivity and actions. We aimed to explore the experience of nurses regarding negative movements toward unethical behaviors. Explaining the process and factors relating to these negative changes can help policymakers and nursing education planners to develop and execute suitable strategies.

## Methods

This was a qualitative study utilizing the inductive thematic analysis approach, which was conducted from February 2017 to September 2019. Thematic analysis is a popular method of qualitative data analysis, and it can show patterns in data [34].

### Setting and participants

The participants were recruited from four university-affiliated hospitals in Tehran (Capital of Iran) and Rasht (Capital of Gilan Province, Iran). Inclusion criteria were having a nursing degree (at least BSc), and work as a practitioner for more than 2 years. Purposeful sampling was used at first to find nurses who have experience with moral decision making. Further recruitment was based on the emerging codes during the data analysis process. Maximum variation was considered based on age, education level, work experience (years), type of hospital (Two generals, one burns, and one cardiovascular) and type of ward (Emergency department, Medical-Surgical, burn, pediatric, ICU, CCU, and plastic surgery). The summary of the characteristics of study participants is presented in Table 1.

**Table 1:** The summary of characteristics of study participants

Variable	N
Gender	
Mal	7
Female	18
Age (years)	
> 35	13
35 <	12
Work experience (years)	
2–10	11
10–20	14
Education level	
Bachelor of science	19
Higher degree	6

The first author chose the first three participants among her co-workers and based on their job history and having enriched experience in nursing moral decision making. Only three participants were colleagues, but for them, like other participants, building trust and holding space for a truthful relationship were strategies that the researcher used to manage the situation. After the emergence of initial codes and tentative sub-themes, new participants were recruited. For example, one participant was recruited because she claimed that she have been punished for moral misconduct. After twenty-three interviews, all existing sub-themes properties were identified, and no new sub-theme was formed and we assumed that the data saturation was achieved. After that two more interviews were conducted to ensure reaching data saturation.

### Data gathering

We used semi-structured in-depth interviews as the main method of data gathering. Each participant was invited by the first author (a Ph.D. candidate in nursing) to study in person. The aim of the study was explained to each participant individually, and written informed consent was obtained. The time and place of the interviews were determined by participants. The first interviews were conducted based on an interview guide which was developed by study team members. The research team consisted of a nursing Ph.D. candidate with seventeen years of experience as a clinical nurse, two nursing professors who are experts in nursing ethics and qualitative research, and an assistant professor who works on nurses and nursing student's moral development. The primary interview guide consisted of three sections, including open general questions, intermediate and ending questions. Open general questions were used for the beginning of the interview, for example, “what are the moral aspects of your daily nursing works?” or “explain your decision makings experience in morally challenging situations?”). The interviews were followed with intermediate questions such as “How did you reach that decision?”, “Why did you act like that?” and “which factors influenced your action?”. Interviews were concluded by ending questions such as “Do you have anything to add?” or “is there something which you did not think about before this interview?”. Following and probe questions such as “can you give me an example?” and “can you explain more?” were used to clarify the statements of interviewees. The interview guide changed according to data analysis, and new questions emerged. For example, to identify the impact of role models, we asked this question, “how your co-workers and role models influenced your moral actions?”. Interviews last between 30 and 70 min. Each interview was listened several times by the first author and then transcribed (See Supplementary, Additional File 1).

### Data analysis

Data were analyzed using an inductive thematic analysis approach developed by Braun and Clarke [34]. They introduced the thematic analysis as a method for identifying, analyzing, and reporting patterns (themes) within data. In the first phase, the first author familiarised herself with the data through immersion by repeated reading of interviews’ transcription sand searching for meanings. The second phase was generating initial codes. An initial list of ideas about meaning units (whole interview, paragraphs, or sentences) was formed and each idea was labeled by a code. Initial codes were developed by the first author. Code labels were discussed in regular meetings by research team members. The third phase was searching for themes. In this phase, potential themes were formed by sorting and connecting relevant codes. Tentative themes were formed by the first author and discussed by all authors in meetings. In the fourth phase, tentative themes were refined and formed the main theme with four sub-themes. Sub-themes and the main theme were named in the fifth phase. Names were discussed and changed several times in research team meetings to reach an agreement over them. The final report was developed as this article in the sixth phase. Representative codes and quotations were chosen to include in the report.

#### Rigor and trustworthiness

Lincoln and Guba's four trustworthiness criteria [35] were used. To achieve credibility, prolonged engagement was used through immersion in data, dedicating enough time for data gathering and analysis, constant comparison of data with data, data with codes, codes with themes, and themes with each other. Member checks and peer checks were used to increasing dependability. Transcribed interviews and codes were checked with participants. The final theme and sub-themes were discussed with one participant. Coding, finding patterns, and naming the main theme and sub-themes were conducted under the supervision of all team members. We limited the initial literature review to decrease the effect of preconception on our analysis and results. To increase confirmability, three audit sessions were held with four external evaluators. A complete description of the research process was used to increase transferability.

## Results

The main theme that emerged from our analysis is moral neutralization. It means that nurses began with ethical practice, but their work environment discourages them. The unethical practice became normal for them. This process makes them give up eventually, and they began to practice unethically. They start to justify their immoral practice. Moral indifference develops in some of the nurses. We named this process, which started with feeling discouraged from becoming morally indifferent as moral neutralization. We found five sub-themes regarding moral indifference including (1) feeling discouraged, (2) normalization, (3) giving up, (4) becoming a justifier, and (5) moral indifference. The main theme, sub-themes, and codes are presented in Table 2.

**Table 2:** Main theme, subthemes, and codes of moral neutralization

Main theme	Subthemes	Codes
Moral neutralization	Feeling discouraged	Be criticized for ethical performance Being punished for doing work ethically Weak encouragement system False judgment Low job satisfaction
	Normalization	When in Rome, do as the Romans do Following neutral role models Doing the orders Earning positive results from wrong actions
	Giving up	Disappointment Stop trying Doing the job wrong Closing eyes on others fault
	Becoming a justifier	Using economic aspects as an excuse Having too many responsibilities as an excuse Rationalaising immoral actions with a heavy workload
	Moral indifference	Decreasing sensitivity Not caring about the patient Not feeling guilty Putting the job first

### Feeling discouraged

The first sub-theme of moral neutralization was feeling discouraged. Nurses started their work after graduation with ethical values that they learned at school. However, they were criticized and punished for doing ethical practice. They believed that the weak encouragement system was the main cause of this feeling.

Some nurses mentioned that they were criticized for ethical performance. When a nurse wants to do things ethically, she/he may be criticized by colleagues and managers, because working this way usually takes more time. For example, while teaching needed cares to the patient's family is the nurse's duty, providing proper care and preventing nosocomial infections takes precedence. Heavy workload and nursing shortage may make some nurses want to lighten their work. They may criticize a nurse who does her/his job in accordance with ethical principles because it is more time-consuming.

In my previous ward, nurses asked the patient’s family to do the endotracheal suctioning. Their excuse was that the family should learn how to do the procedure because they have to do it in the home. But I did the procedure myself. They criticized me because I was disturbing their routine (Nurse 16).

Being punished for doing work ethically was a pattern that we found in the data. In some hospital wards, doing things immorally has become routine. In these wards, newcomers who want to work with the ethical principles that they have learned are suppressed by the managers. Department managers do not tolerate this kind of work because they will change their routines.

In our ward, preparing the deceased for transfer to the mortuary is done by environmental services and the patient’s family, while it is the responsibility of the nurse. The first time I did it for a patient, the head nurse wanted me and penalized me harshly. She said you do not have the right to change our way of doing work (Nurse 21).

Nurses blamed the weak encouragement system for this feeling. When a nurse does her job ethically, she/he expects the system to encourage her/him. Some hospitals do not have a system that encourages ethical performance. Lack of a proper encouragement system along with punishment for ethical work reduces the moral motivation to act.

The election of the most ethical nurse of the year is based on work experience. It doesn’t matter how ethical you are; you should wait until your turn comes (Nurse 6).

Some nurses thought they were falsely judged. In a context where the unethical practice has become the routine, false judgments were the result of confusion between ethical practice and being slow or a novice. In such a situation, it becomes difficult for the nurse to have moral performance.

Because I do nursing care very carefully, it takes a lot of time. My work usually lasts long after the shift. My colleagues have accused me of being slow (Nurse 8).

Being criticized or punished for ethical work led to low job satisfaction. A patient with poor condition sued a nurse over his pain while the nurse put analgesic on hold because of its side effects. Lack of enough support for working ethically can lead to job dissatisfaction.

I put the patient’s analgesic on hold because it started to show side effects. He had pain yet, but the side effects were hypotension and bradycardia which are dangerous. He sued me, and no one supported me for that. I did the procedure correctly. A situation like this makes you unhappy (Nurse25).

### Normalization

Nurses start to do what they see and learn from the work environment and other nurses, things they order to do. Furthermore, they earn positive feedbacks and result from unethical practices.

Some nurses, after entering the profession, tried to be like others to avoid confrontation. They said that when in Rome, do as the Romans do. In this case, the nurse may imitate other immoral actions to be like everyone else.

In our ward, it is routine to infuse several drugs together. This makes things run faster. I do it like others, to finish my work more rapidly (Nurse15).

Role models play an important role in shaping the moral performance of nurses. Many role models affect the nurse after entering the profession. If the nurse follows role models that are morally neutral or negative and have immoral performance, it will have a negative impact.

When I was new to this ward, one of my colleagues who had 15 years of work experience, was my model. Once, a patient had a cardiac arrest, he did not CPR the patient. Only he shook the leads to have a CPR strip on the monitor. After that, I learned to do the same on patients who need CPR (Nurse12).

Many novice nurses follow the ward’s managers and centers of power. The hierarchy of power and work experience that exists in nursing makes junior nurses follow senior nurses. Our participants told us that they began to do the orders after a time because they did not have enough authority.

Our head nurse does not believe in administering opioid analgesics to addict patients. She says it is a waste. She forbids us to administer opioids to them. We do the order regardless of the severity of the patient’s pain (Nurse 5).

Rewarding immoral work can cause people to repeat it. For example, early completion of work or approval by a superior can cause a person to repeat his or her immoral work. Some nurses argued that they stopped doing the right thing because they earned positive results from wrong actions.

Before doing the postoperative care, I had it recorded in the report. Supervisor arrived and only read my report without checking the patient. She applauded me for writing such a detailed report without checking the patient. I realized that good care is not important, we should only write it down. I was punished before, for a late recording of the care that I have been done (Nurse 17).

### Giving up

Eventually, some nurses become disappointed, they do not attempt to do the work ethically, and they may start doing immoral actions.

Getting negative results from moral performance and positive results from immoral performance and the lack of an accurate and appropriate encouragement and punishment system caused the nurse to give up. Some nurses express their disappointment from doing ethical practice.

There is no point to do work ethically, nothing will change. I tried it by reporting the faults in the ward, and nothing happened. I am so disappointed with the system (Nurse 10).

When nurses did not get a positive result from their ethical work, they did not continue. One of our participants said she stopped trying to do the right thing because she thought it is futile.

Previously, I used to report medical errors of my colleagues. However, I stopped it, because the head nurse did not care or handled it, a way that caused a lot of problems in the ward (Nurse 20).

The positive consequences of the immoral act prompted the nurses to start doing it. This positive result may have happened to them or they may have seen it in the work of others. Some nurses mentioned that they started to do the job wrongly.

We are ordered to record ECG every three hours. I take all three ECGs on one occasion, and I write a three-time stamp on them (Nurse 15).

### Becoming a justifier

Nurses who we were interviewed tried to justify their immoral actions. They used economic problems, having too many responsibilities, and a heavy workload as excuses. The following quote is an example of using economic aspects as an excuse.

Bathing the patient is the duty of the nurse. I used to do it myself. But now I give it to environmental services. How much they pay me to do such hard things (Nurse 6).

Nurses who still cared about moral performance tried to justify their immoral actions. Some nurses claimed that they had too many responsibilities, and this forced them to do immoral actions.

I have a lot of paperwork. How do I suppose to provide good care of my patients, talking to them, or providing mental health care for them? I have too many responsibilities (Nurse 21).

Numerous reasons such as high workload, low wages, nursing shortage, high responsibilities, and low working hours were explanations that were used to justify immoral actions. Nurses were trying to calm their conscience in this way. The following quote is the rationalization of immoral actions with a heavy workload by an interviewed nurse.

When our ward has too many turns over, I administer the antibiotics with intravenous injection, I don’t have time to do the infusion (Nurse 7).

### Moral indifference

Some nurses reach a level of moral indifference, which shows itself with a decrease in sensitivity to the moral aspect of care. They even stopped trying to justify their immoral action and put morality away. Decreasing sensitivity was one of the main aspects of moral indifference which was mentioned.

When I came to this ward when the physician asked me to decrease the ventilator support, it was very hard for me, I couldn’t do that, it was very challenging. Now, I do that without thinking about it. I will do the order (Nurse 2).

Levels of moral numbness may occur in the nurse. Because the nurse does not care about moral performance at this stage, she/he may even intentionally do immoral work. One of the nurses mentioned that she was not caring for the patients anymore.

Sometimes you don't’ know something, and you do a procedure incorrectly. But I have enough knowledge. For example, I know that the restrictions shouldn’t be tight, and we have to check them several times during the shift. But I don’t do that. I don’t care (Nurse 12).

In some cases, nurses who initially tried to engage in ethical practice became so immoral that they deliberately committed immoral acts. Some of them were even proud of committing immoral acts. Some of them said that they lost their feeling of guilt.

When I was a novice, I used to administer the drugs on time. When I didn’t do that I had a guilt feeling. Now, I administer all drugs at the beginning of the shift without looking at their time of order, and I don’t have any bad feelings about it (Nurse 19).

Finishing daily work without even paying attention to the ethical aspects had become a priority for some nurses. One of our participants mentioned that she put the job first without considering moral aspects.

While I am at work, most of my attention is on completing the job. I don’t think about patients’ feelings and even their health. For example, when I fix the IV, I use a lot of surgical tapes. I want it to be fixed completely and making no problem for me. It can hurt the patient's skin, but it doesn’t matter. What I care about is only doing the job (Nurse 23).

## Discussion

The current study aimed to understand how some nurses' ethical performance changes negatively during their work years. Our findings indicate a pattern of neutralization in nurses’ moral practice. The pattern includes five components, including feeling discouraged, normalization, giving up, becoming a justifier, and moral indifference.

Moral neutralization is a known theory in criminology, which was introduced by Sykes and Matza [36]. They argued that wrongdoers learn neutralization techniques, so they can break the laws and norms they believe in, in this way the offenders protect their self-esteem. In comparison with this theory, our results showed that nurses who have immoral performance defend themselves by arguing that their immoral actions are acceptable under current circumstances [36]. Our results showed some aspects of neutralization in nurses’ immoral actions and their justifications. While the theory is about criminal actions, and most of the nurses’ immoral actions do not have criminal nature, the effect of moral climate, normalization, and using justification are three major similarities between our model and moral neutralization theory. Based on our results some level of normalization happened in nurses who have immoral performance. Nurses with immoral performance use unethical climate for normalization of their behavior and excuses for justification.

The environment plays a key role in immoral practice and its subsequent justification [37]. Our results emphasized the role of the moral culture of the healthcare organizations on the ethical practice of nurses. With a nursing shortage in the Iranian health care system, [38], nurses’ workload has increased. The shortage of medical equipment and lack of appropriate auditing are the other problems that Iranian nurses have to deal with in the health system[39].

The experience of the nurses indicates that feeling discouraged plays an important role in moral neutralization. Encouragement creates a satisfying feeling and stimulates the individual's motivational system. The effect of moral discouragement on immoral actions can be explained by the learning theory of Thorndike. Based on this theory, if a behavior results in an unpleasant experience, that behavior will not be repeated [40]. Our results showed that the punishment or not being acknowledged after moral actions discouraged nurses to act ethically. Previous studies have shown that encouragement in a health system plays an important role in inspiring nurses to perform ethical practice and vice versa [41, 42]. The work-place ethical climate is an overarching concept for the approaches that organization leaders use in treating their staff. Critical to nurses’ performance is the presence of a positive ethical climate—“the implicit and explicit values that drive health-care delivery and shape the workplaces in which care is delivered” [43]. Even though studies conducted over several years in diverse areas of nursing practice have shown that nurses face serious ethical challenges, researchers and policy-makers who study the work environment use an ethics lens to look at workplace problems [44]. Previous research has shown that the moral climates of Iranian hospitals are not encouraging, and the reward and punishment systems did not support moral practice [42, 45–47]. In this situation, nurses cannot recognize and solve moral issues [48, 49]. In this moral climate, immoral actions become normalize in time.

Our results showed that some level of normalization of immoral actions has happened in nurses. This normalization can be explained by Gidden's theory of structuration. Based on the structuration theory, normalization of interaction patterns is the factor that makes them persistent over time [50]. Based on our results normalization of immoral actions in healthcare systems occurs through a lack of conscious thought about their rightness. It is in line with previous studies that showed normalizing teaches newcomers to accept and do immoral acts [51]. In the unethical climate of hospitals, our participants tried to avoid being misjudged by modeling other nurses who were successful in this climate.

Based on our results novice nurses have the potential to be influenced by the behavior and attitude of their colleagues. Our results showed that this influence can be positive or negative. Novice nurses try to copy the performance of their colleagues and to be like them. In situations that they do not have enough knowledge, they are trying to emulate their experienced colleagues to improve their nursing skills [52]. Sometimes they may learn negative behaviors [53]. stated that nurses, instead of having evidence-based practice, work based on their colleagues’ experiences [53].

In an unethical climate, nurses may give up moral performance and even start to engage in immoral actions. This result is consistent with previous studies. For example, the results of Zabuli et al. (2015) showed that studied nurses believed that trying to have moral performance is futile, and they were not trying to work ethically [54]. Peyravi et al. 2016 resulted that there were four major barriers to the reporting of errors by Iranian critical care nurses, including (1) saving professional reputation and preventing stigma, (2) fear of consequences, punishment, and legal problems, (3) feelings of insecurity and lack of managerial support and (4) not investigating the root cause of the error [55].

When a nurse commits an immoral action, he/she tries to justify that. Justification is the main part of immoral actions. Furthermore, moral justification is the first of a series of mechanisms suggested by Bandura. He argued that moral justification can induce people to bypass self-sanction and violate their standards[56] following study showed that instead of using ethical and critical reasoning or pertinent theoretical bases nurses tend to decide and justify their moral actions by calling on their values and experiences. They concluded that nurses’ ethical decisions are based mainly on personal principles, religious beliefs, their previous education, intuition, and feelings or group desires [56]. Our results showed that nurses justified their immoral actions by workload, the chain of order, and organizational culture. The results of Zhao also referred to moral justification as sanitizing the act, which involves portraying the act as commendable, either socially or morally [57]. Hyatt [58] concluded that when clinicians begin to defend their immoral behaviors that impact a culture of safety or resort to sullying patients or families to redirect attention from those behaviors it can negatively impact the institutional culture and individual practitioner [58]. The Moral justification was mentioned in several studies as a mechanism that was used by nurses in order to rationalize their unethical behaviors. The results of a systematic review showed that nurses and physicians used several reasons to morally justify to continue using sedatives at the end of life care [59]. In another study, results showed that nurses justified not reporting of medical errors with trying to maintain their interpersonal relationships [60]. The result of a qualitative study showed that nurses used the heavy workload and not having enough time as two excuses for not doing change positions in immobile patients [61].

Based on our results gradually, some nurses become morally indifferent [62]. defined moral indifferent persons as one who sees the moral situation and has not any sense about it [62]. The results of Choe et al. in 2015 showed that nurses became morally indifferent due to high workload and time constraints [63]. The results of a study on Norwegian pediatric nurses showed that they did not find themselves morally committed to control the pain of children or to use scales to assess the pain [64].

### Limitations

We tried to translate and label our findings to be understandable in different cultures, but we are aware of language limitations, especially in qualitative studies. The Iranian nursing culture and health system's ethical climate may be different from that of other countries but there is a similar pattern in the literature regarding failing health systems to reach a high level of moral standards. Furthermore, we explained our methods and findings in a way that other researchers could transfer this study to their contexts.

## Conclusion

The findings of our study showed that some nurses became indifferent to ethical aspects of care, and they do not attempt to work ethically. Trying to stay ethical in an environment where most people are not sensitive to ethical issues is difficult. This will ultimately cause stress and burnout for the nurse. Feeling discouraged is the external reason that affects nurses' moral practice. Based on our results being punished or criticized for ethical performance, falsely judged for doing the job right can lead to low job satisfaction. Overtime, unethical practice becomes normalized for them, and they give up in front of the job environment. They try to calm their conscience by justifying their actions. In the end, some of the nurses lose their sensitivity toward moral aspects of their actions, and moral indifference will be developed in them. This is the process of moral neutralization that we found in our participants.

While our results showed that negative changes may happen in nurses, most nurses work with the highest level of moral standards around the globe. Policymakers in the health systems can benefit from our results in the development of their strategies. Hospital managers should consider effective reward and punishment systems to deal with moral issues. Also, they should consider ethical aspects in all of their decisions. Managers in the education area may use more suitable content and strategies to empower nurses in the field. One implication of our results is that nurses need to be involved in institutional and perhaps broader policy changes. Since the organization's ethical climate affects nurses' ethical performance, nurses should be trained to act as change agents rather than the target of change. The educational curricula should prepare nurses for taking the new roles and fighting for creating an ethical climate in their workplace. It is suggested that ethical practice receive more attention in system, organization, and unit level, by considering the ethical performance as part of nurses’ evaluation system.

## Supplementary information

**Additional file 1**. Interview Guide. Explaining moral development among nurses: a qualitative research.

## Data Availability

Not applicable.
