# Supplementary material for: Moral neutralization: Nurses’ evolution in unethical climate workplaces
Source: BMC Med Ethics. 2020 Nov 17;21:114. doi: 10.1186/s12910-020-00558-3 (PMC7672869; doi:10.1186/s12910-020-00558-3)
Supplement: Supplementary file 1 — Additional file 1. Interview Guide. Explaining moral development among nurses: a qualitative research. [file 12910_2020_558_MOESM1_ESM.docx]

**Interview Guide**

**Explaining moral development among nurses: a qualitative research**

**Introduction:** As you know, and probably you encountered with, in profession of nursing, you will frequently face with situations that need to decide between right and wrong action. In ordinary life, also, we think about our actions. If, you look to the past, in several situations, you might think about your actions and your decisions. Now, if you think about your decisions, you may remember, why you made that decision and what was your reasons. In a same way, in nursing, when you face with such situations you will think about that, was my work right or wrong but you may not think about your reasons and if your decision was right based on what? In these research, we are interested in asking you about your decisions and the reasons behind them. We want you to think about your decisions? And your reasons for them?

**Initial Open-ended Questions**

1. What are the moral aspects of your daily nursing works?
2. Explain your decision makings experience in morally challenging situations?
3. Have you ever experienced that you stuck between doing right or wrong? Can you describe -------? What happened when ----?
4. What ----- was like? What did you feel when ----? Can you describe, if you remember, what you think when ---? What made you to do that? Or made that decision?
5. If you remember, what motivated you to do ----? Or make decision about -----?
6. What kind of person were you when you did ----? How you think about moral issues?

**Intermediate Questions**

1. How did you reach that decision?
2. Why did you act like that?
3. Which factors influenced your action?
4. In situation like ----, do you like to have who around you? Usually you ask from who for help or support? What kind of help? For what kind of help? Who can help you in this situation better?
5. Do you like to face --- again? If you will face ---- again, which decision will you make? What do you will do?
6. Did you ever think about your decision? What was your reason for?
7. Do you ever felt that you have trouble to decide about another person act? When was ---?
8. What happened that you did ----? Who had influenced on ----? Who they had influence on ----?
9. Can you explain the events that lead to ----? What was the reason you did ----?
10. Did the same thing happen before? What did you do the last times? Have it happened to a person you know?
11. How do you manage situation like ----? You learned ----- from who?
12. What changes take place from ---- in your decisions about the right and the wrong? Which one was positive or negative?
13. How strong are you in facing moral issues? How were you before? Better or worse? How? Did something else happened (like marriage, meeting somebody, loss of someone or traveling to someplace) which influenced on this changes?
14. What lesson you learned from ---?
15. What kind of person do you like you was when ---? What kind of person do you like to be? Since the beginning of your education what differences occurred to you?
16. What problems or obstacles there are in the way of making positive changes in nursing students?
17. Who have the most effective influence on these changes? Who or what can contribute on these changes?

**Ending Questions**

1. Do you have anything to add?
2. Is there something which you did not think about before this interview?
3. What changes happened in your idea about right and wrong since college?
4. What do you recommend to people who may face to same experience?
5. Is there something else you think I should know to understand _______ better?
6. Is there anything you would like to ask me?
